# Enhancement of Radiation Effectiveness in Proton Therapy: Comparison Between Fusion and Fission Methods and Further Approaches

**DOI:** 10.1038/s41598-020-62268-5

**Published:** 2020-03-25

**Authors:** Farshid Tabbakh, Narayan S. Hosmane

**Affiliations:** 1 Nuclear Science and Technology Research Institute, Tehran, Iran; 2 Department of Chemistry and Biochemistry, Northern Illinois University, DeKalb, IL 60115-2862 USA

**Keywords:** Cancer therapy, Computational science, Nuclear physics

## Abstract

Proton therapy as a promising candidate in cancer treatment has attracted much attentions and many studies have been performed to investigate the new methods to enhance its radiation effectiveness. In this regard, two research groups have suggested that using boron isotopes will lead to a radiation effectiveness enhancement, using boron-11 agent to initiate the proton fusion reaction (P-BFT) and using boron-10 agent to capture the low energy secondary neutrons (NCEPT). Since, these two innovative methods have not been approved clinically, they have been recalculated in this report, discussed and compared between them and also with the traditional proton therapy to evaluate their impacts before the experimental investigations. The calculations in the present study were performed by Geant4 and MCNPX Monte Carlo Simulation Codes were utilized for obtaining more precision in our evaluations of these methods impacts. Despite small deviations in the results from the two MC tools for the NCEPT method, a good agreement was observed regarding the delivered dose rate to the tumor site at different depths while, for P-BFT related calculations, the GEANT4 was in agreement with the analytical calculations by means of the detailed cross-sections of proton-^11^B fusion. Accordingly, both the methods generate excess dose rate to the tumor several orders of magnitude lower than the proton dose rate. Also, it was found that, the P-BFT has more significant enhancement of effectiveness, when compared to the NCEPT, a method with impact strongly depended on the tumor’s depth. On the other hand, the advantage of neutron risk reduction proposed by NCEPT was found to give no considerable changes in the neutron dose absorption by healthy tissues.

## Introduction

In addition to using the radionuclide as an internal radiation source for treatment of tumors in nuclear medicine, there are other kinds of modalities existent based on radiation arising from external sources^1^. These modalities are X-ray from medical electron accelerators, gamma-knife using gamma-ray^2^, boron neutron capture therapy (BNCT)^3–8^ involving thermal neutrons and ion therapy or hadron therapy using ions like carbon and the proton^9–18^. In BNCT (which is currently being investigated), the stable isotope of boron ^10^B readily captures neutrons, while the ^11^B isotope does not. Biomolecules and drugs containing ^10^B‐enriched moiety (which preferentially localizes in tumor cells and rapidly clears from normal cells) can thus be used for cancer therapy. When the ^10^B nuclei are bombarded with thermal or epithermal neutrons, they undergo a fission reaction which produces high energy alpha (α) particles and ^7^Li ion, both carrying the released fission energy. Since these α particles travel only about 10 µm or less, they selectively destroy cancer cells where the ^10^B nuclei are localized^3–8^. Another modality with higher radiation effectiveness is proton therapy^10,11^ in which the high energy protons penetrating the target able to make damage in the cancerous cells effectively. In proton therapy, the proton particles (and charged particles in general) in passage through matter will finally stop and deposit their remaining kinetic energy at the stopping position defined by the Bragg’s peak concept^14^. This leads to an important fact that, the incident beam can be controlled and can be stopped at the desired point within the tumor. This controllable therapeutic process beside the clinical conditions, have made the proton therapy a more effective therapeutic method than gamma-ray^9^. The enhancement in radiation effectiveness in proton therapy is one of the main subjects in many studies and, recently, the Proton-Boron Fusion Therapy (P-BFT) as a newly proposed method in proton therapy has attracted much interest^19–21^. Fusing protons into boron-11 will release three high LET alpha particles, giving enhancement in effectiveness of proton therapy and causing more damage to the cancerous cells. Another newly proposed method to enhance the radiation effectiveness is Neutron Capture Enhanced Particle Therapy (NCEPT), which uses boron-10 agent to capture the low energy secondary neutrons^22–26^ during the proton therapy^9^.

Since neither method has been tested clinically, the present calculations reveal the impacts related to the P-BFT and NCEPT methods and made a comparison with respect to different concentrations of boron isotopes for different tumor depth or Bragg’s position to evaluate the effectiveness of P-BFT and NCEPT.

In addition to this comparison, an evaluation was performed to investigate the impact of the NCEPT method in reducing the neutron dose rate to the healthy tissues (neutron risk to patients^27,28^) which was proposed as an advantage of this method.

In this report the calculations were conducted using the Monte Carlo methods. Although, the Monte Carlo methods are not able to precisely simulate the problems corresponding to the conditions in the real world, in studying the particles passage through the matter and nuclear interactions have been widely used as powerful and accurate tools in many applications of radiation researches, such as the ion therapy studies^29–37^. Thus, to obtain the most reliable results, the two Monte Carlo tools, the MCNPX introduced by Hughes, *et al*.^38–40^ and the GEANT4 by Agostinelli, *et al*.^41–44^ were used. A detailed description of the methodology in the present report is presented in the Methods Section, which is then followed by the Results and Discussion Section.

## Results and Discussions

**Figure 1:**
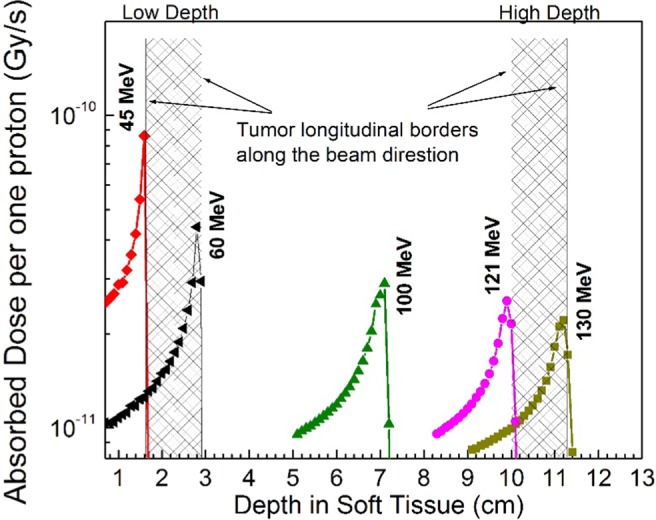
The SOBP corresponding to the tumors at two positions; low depth (near the surface) and high depth in the spherical phantom of the head, calculated by GEANT4. It shows the tumor’s longitudinal borders with the corresponding Bragg’s peaks and the proton energies.

In the present study, the depth of the tumor is an important parameter for the later results and discussions, Fig. 1 using the GEANT4, illustrates the proton beam with two energy ranges and the corresponding SOBP for the low depth and high depth tumors respectively. As it is shown, the tumor with 1.4 cm of diameter has been treated by the proton beam with spread energy from 45 MeV to 60 MeV for low depth position (SOBP from 1.6 cm to 3 cm) and 121 MeV to 130 MeV for the deepest position (SOBP from 10 cm to 11.3 cm). The calculated energy ranges and the corresponding Bragg’s peaks illustrated in this figure have agreement with the other reported data^25,26,45^. In the following presented results, the Bragg’s end position and the corresponding proton energy have been mention to represent the tumor’s depth.

The following results have been organized into different subsections; the results of the NCEPT method evaluations, the impact of NCEPT in reducing the neutron absorption dose to the surrounding tissues and the results of the P-BFT method evaluations. A more comparative discussions have been presented at the of this section.

### Evaluation of the NCEPT

**Figure 2:**
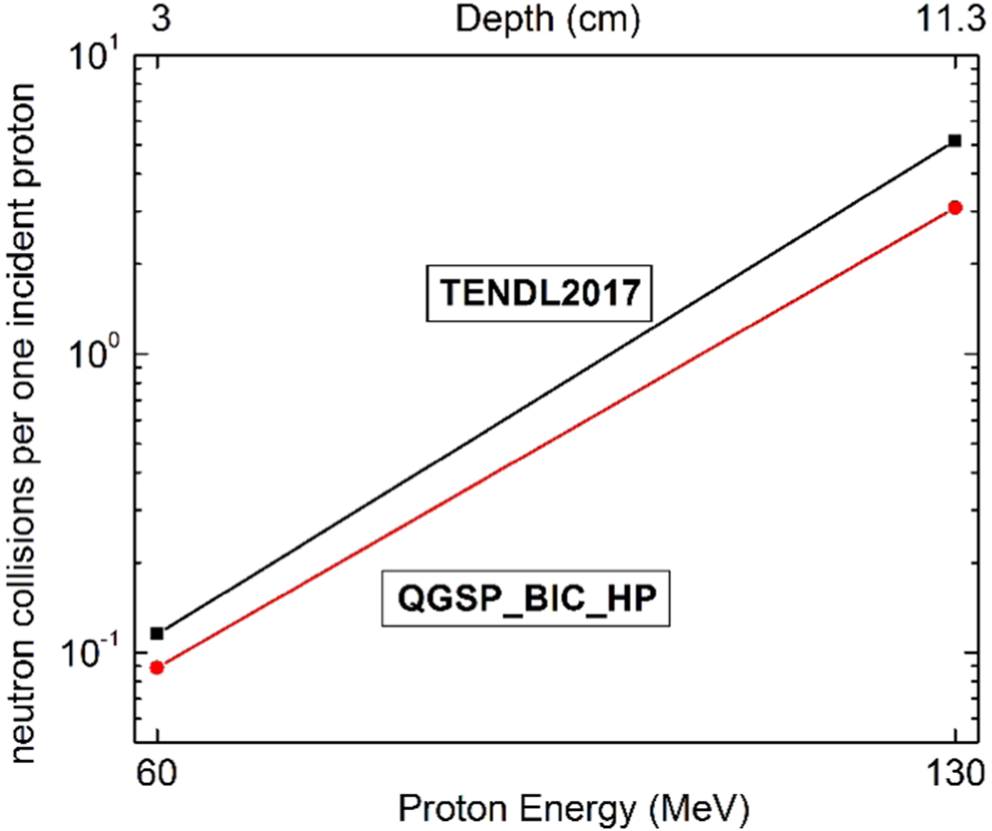
The normalized neutrons collision per one incident proton in terms of depth of the tumor calculated by MCNPX and GEANT4. Both the MC methods show almost two orders of magnitude increase in the number of the collisions before reaching to the tumors at deeper position comparing to the number near the surface. This is an evaluation of the impact of the geometry in the neutron related treatments.

Since the secondary thermal neutrons play the main role in NCEPT, an overview of the secondary neutrons collisions leading to the thermalizing process has been calculated before studying the neutron flux inside the tumor. Figure 2 represents the total number of the neutron collisions (per proton) in terms of the tissue’s depth and the corresponding protons energies obtained from MCNPX and GEANT4 respectively. The proton beam with maximum energies of 60 MeV and 130 MeV are corresponding to Bragg’s end position at 3 cm and 11.3 cm respectively as described before. As anticipated, the higher number of the collisions in the figure refers to the higher depth (SOBP 10 cm to 11,3 cm). Both the MC results show that, as the proton energy increases, the number of produced neutrons has been increased^26^ and also, the deeper position means the more moderating medium for neutron slowing down. From this figure, one can predict the higher value of low energy neutrons corresponding to the deeper tumors in compare to the tumor at the lower depth. This figure gives a background about the impact of the geometry in the neutron related treatments.

**Figure 3:**
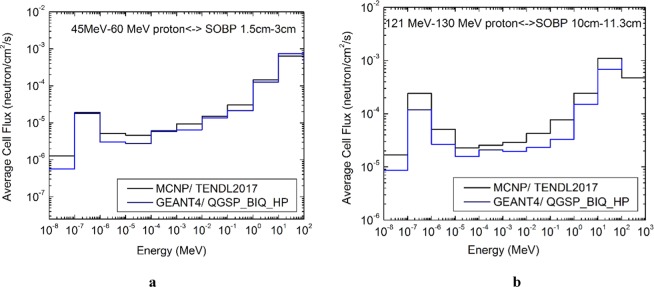
The average secondary neutrons spectra in the tumors at two different depths obtained by GEANT4 and MCNPX. Two incident protons energy ranges have been examined; **(a)** 45 MeV to 60 MeV for the low depth tumor with SOBP from 1.6 cm to 3 cm which shows the thermal neutrons with the flux much lower than deeper tumor presented by **(b)** 121 MeV to 130 MeV protons entering to the deeper tumor (with SOBP from 10 cm to 11.3 cm) showing significant increase in the thermal flux. The two MC tools presented the relatively strong dependency of the thermal fluxes on the tumors depth. This figure, also shows the large contribution of the fast secondary neutrons in proton therapy.

The prediction of the dependency of the low energy neutrons on the Bragg’s peak already performed by Fig. 2. The detailed evaluations have been confirmed by the two average fluxes presented in Fig. 3a,b. These figures depict the normalized neutron spectra inside the tumor at two previously described positions. The results were obtained from both the MC tools and compared to perform a more reliable evaluation. As it is shown in Fig. 3a the thermal flux around $1.5\times {10}^{-6}\frac{n}{c{m}^{2}s}$ by MCNPX and even lower value by GEANT4 near $8\times {10}^{-7}\frac{n}{c{m}^{2}s}$ for the SOBP between 1.6 cm to 3 cm. By increasing the depth, as presented in Fig. 3b, the average thermal flux rises to $1.8\times {10}^{-5}\frac{n}{c{m}^{2}s}$ and $9\times {10}^{-6}\frac{n}{c{m}^{2}s}$ according to MCNPX and GEANT4 respectively. Before proceeding to the reaction rate calculations, here, a validation has been performed based on the other published researches. Several works have been presented showing the neutron flux variation as a function of the position (Bragg’s peak) or the proton energy. For example; Schneider, U. & Halg, R^23^ have demonstrated the neutron dose equivalent as a function from the distance of the field edge. Also, Wei, J. Y. J^26^. has performed a comparison between 70 MeV to 200 MeV proton beams and has reported that, the neutron counts varies from 10^3^ to 10^5^ respectively. Similarly, Jia, S. B., *et al*.^25^ showed the neutron production as a function of the proton energy which varies from 0.27% to 10.14% corresponding to the proton energies from 40 MeV to 140 MeV respectively or two orders of magnitude which confirms the Wei, J. Y. J. results.

These figures also show that, the epithermal fluxes, vary from ${10}^{-5}\frac{n}{c{m}^{2}s}$ to ${10}^{-4}\frac{n}{c{m}^{2}s}$ from the low depth to higher depth respectively. Though, from these figures, the GEANT4 has relatively lower values than MCNPX but, the main results show the compatibility of the two MC results in variation of the secondary neutrons (all energy ranges) in terms of the incident protons energy and the depth in tissue.

Another important fact depicted by these figures is the large contribution of the fast neutrons with the maximum energy up to the incident protons energies. The flux of the fast neutrons reach to almost ${10}^{-3}\frac{n}{c{m}^{2}s}$ independently of the depth. This is important in evaluating the absorbed dose rate by surrounding healthy tissues from the secondary neutrons, as discussed later.

After the neutron spectrum in tumor was derived, as the second step, the ^10^B isotope has been added to the tumor (Table 1). To evaluate the NCEPT maximum impact under a realistic concentration of ^10^B, the present work performed the calculations for concentrations of 100 ppm. The higher concentration up to 1000 ppm have been examined later as a further evaluation of NCEPT impact comparing to the traditional proton therapy.

**Table 1:** The important parameters and information related to GEANT4 and MCNPX setup, the geometry and the materials, the proton beam.

GEANT4	Physics list:	QGSP-BIC-AllHP for (P + ^11^B) QGSP-BIC-HP
	Particle data libraries	PARTICLEHPDATA (G4TENDL1.3.2)
MCNPX	Proton and neutrons data libraries	TENDL 2017
Geometry and Materials	The tissue’s elements mass fractions^25^	Carbon: 20%, Hydrogen: 10%, Oxygen: 65%, Nitrogen: 5%
	Phantom (Human’s head)	Sphere with the radius of $9\,cm$ consists of soft tissue, ($1.1\frac{g}{c{m}^{3}}$).
	Tumor	Sphere with the radius of 0.71 cm / the volume of ~1.5 cm^3^, ($1.1\frac{g}{c{m}^{3}}$).
Proton beam	proton beam diameter	2 mm
	Beam Energies corresponding to SOBP (Figure 1)	45 MeV- 60 MeV $\leftarrow \to$ SOBP: 1.6 cm – 3 cm 121 MeV - 130 MeV$\leftarrow \to$ SOBP: 10 cm – 11.3 cm
Boron isotopes	NCEPT	Only, ^10^B isotope (distributed homogeneously in tumor)
	P-BFT	Only, ^11^ B isotope (distributed homogeneously in tumor)
Dose-Rate calculations	Quality factor used in Eq. 2 corresponding to the alpha particles and heavy nuclei (adapted from NRC)^49^.	Q (alpha and heavy ion s) = 20
	ICRP Weighting factors for heavy ions (W_R_)^49^.	W_R_ = 20
	ICRP Effective Quality factor for the neutrons^50^	Thermal: 2.3, Epithermal: 2, Fast: 6–11
	ICRP Weighting factors for the neutrons (W_R_)^50^.	Thermal: 5, Epithermal: 10, Fast: 5

Also, the quality and weighting factors for heavy charged particles and the neutrons according to the ICRP which, were used in the present dose rate calculations.

In following, the MC tools estimated the reaction rates corresponding to the low and high depths presented in Table 2. According to these results, when the depth increase from 3 cm to 11.3 cm (almost 70% increase) the estimated reaction rates increase almost 90%, from both the MC tools.

**Table 2:** The calculated (n, α) reaction rates in terms of the Bragg’s end points and proton energy.

Bragg’s End point/proton energy	3 cm/60 MeV	11.3 cm/130 MeV	Increase in the reaction rate
MCNPX reaction rate/P	$9.5\times {10}^{-8}$	$1.09\times {10}^{-6}$	91.3%
GEANT4 reaction rate/P	$8.14\times {10}^{-8}$	$7.6\times {10}^{-7}$	89.3%

The two MC tools obtained almost 90% increase in (n, α) reaction rate, when the depth increases 70%.

**Figure 4:**
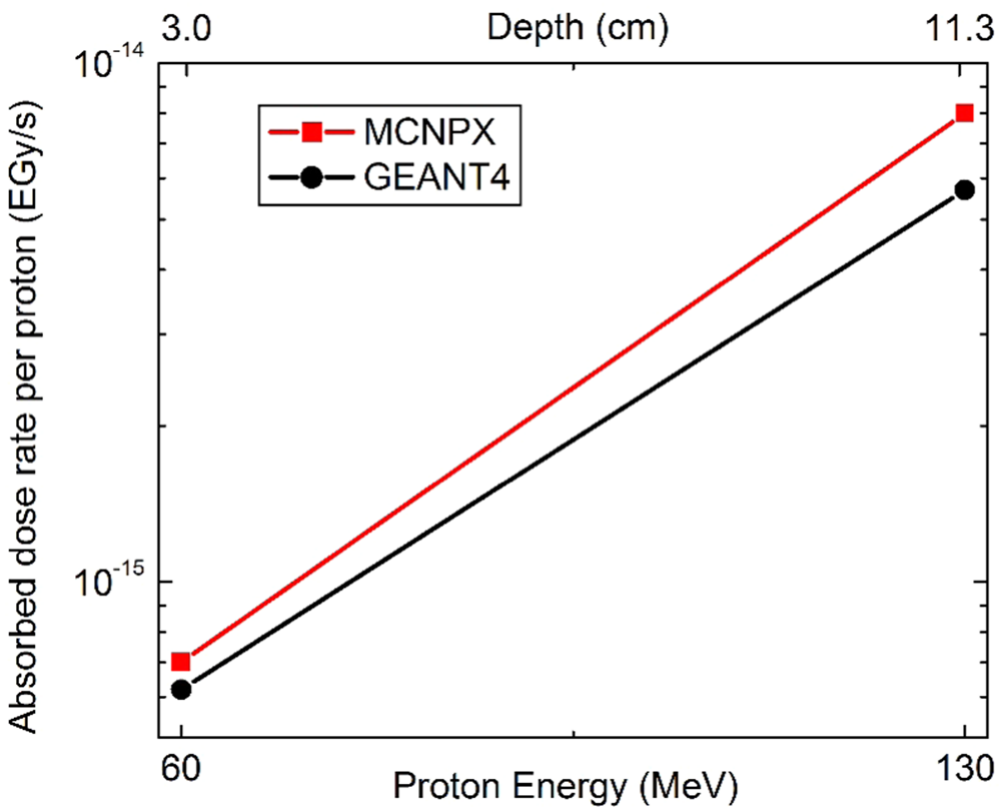
The MC results regarding to the NCEPT, for the excess equivalent dose rate received by tumor (EGy/s) according to the depth of tumor. This figure presents 90% increase to the delivered dose rate in NCEPT, when the depth increase 70%.

Each n-capture with ^10^B releases one alpha particle ($1.5\frac{MeV}{alpha}$) and one ^7^Li ($0.8\frac{MeV}{Li-7}$) ion^3–8^ which have a short range of a few microns. Using the Eq. 2 and the reactions rates presented in Table 2, the corresponding equivalent dose rates have been presented in Fig. 4. In this figure, shows the MC results for the excess delivered dose rate using the NCEPT method in terms of tumors depths when the tumors contain 100 ppm of ^10^B. Accordingly, 70% increases to the depth (from 3 cm to 11.3 cm), leads to 90% increase to the dose rate received by tumor showing that, the effectiveness of the NCEPT is strongly depending on the tumor depth in tissue.

### Investigating the Variation of the Neutrons Absorption Dose Rate in NCEPT

**Figure 5:**
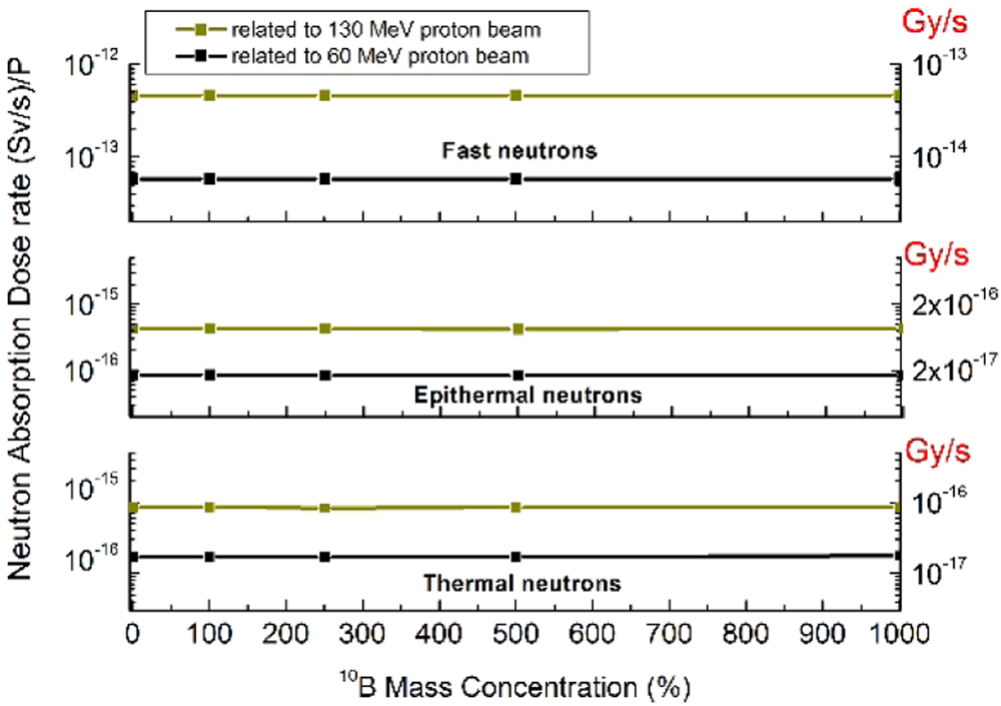
The equivalent absorbed dose rate $(\frac{Sv}{s})$ corresponding to the secondary thermal neutrons (shown by the lowest part of this figure), the epithermal (the middle part) and the fast neutrons (the upper part) during the treatment by proton therapy. The horizontal axis shows the ^10^B concentration increase from 0 ppm (as traditional proton therapy) up to 1000 ppm of ^10^B. These parameters have been presented for two incoming proton energies, 60 MeV and 130 MeV by golden and black lines respectively. The results were obtained by the calculated MCNPX fluxes of three groups of energies and the ICRP flux to dose rate conversion factors. The dose has been presented in $(\frac{Sv}{s})$ as the left vertical axis and also in $(\frac{Gy}{s})$ as the right vertical axis.

As it has been proposed in the NCEPT method, another aspect in using ^10^B is to reduce the risk of thermal neutrons affecting healthy tissues. The following calculations have been conducted to re-investigate the impact of the NCEPT in decreasing the neutrons risk. To this purpose, the equivalent dose rate in healthy tissues from the secondary neutrons have been examined by MCNPX according to the different ^10^B concentrations. In the research performed by Safavi-Naeini *et al*.^9^, the rate of the thermal neutrons reduction has been considered equal to the rate of the capture reactions. But, due to the spatial distribution of the neutrons over the whole geometry in all energy ranges (Fig. 2 also shows this fact that thermal neutrons appear outside the tumor), in this report, this reduction has been investigated in terms of the thermal neutron equivalent absorption dose rate in healthy tissues. Also, from the descriptions of Fig. 3, to perform a more realistic evaluation, the contributions of the epithermal and fast neutrons have been calculated. The calculations were based on the flux-to-dose rate conversion factors released by ICRP. Accordingly, the normalized equivalent absorbed dose rate $(\frac{Sv}{s})$ delivered to the surrounding healthy tissues corresponding to three groups of neutrons for two incoming proton energy have been shown in Fig. 5. In this figure, using the ICRP weighting factors (Table 1), these values converted into the dose rate in $(\frac{Gy}{s})$ have been re-scaled as the vertical axis at the right-side of this figure. As one can see from the lowest and the middle parts of this figure, increasing the ^10^B in tumor up to 1000 ppm will cause no decrease to in the absorbed dose rates. Additionally, the calculations also show that, the distributed ^10^B in healthy tissues is negligible too. The upper part of this figure also shows, a higher level of the equivalent dose rate to surrounding tissues corresponding to the fast neutrons which is 3 orders of magnitude even larger than those of the thermal and epithermal.

### Evaluation of the P-BFT

The following discussion describes the evaluation of the dose rate delivered to tumor by the P-BFT method. As previously described in the Methods section, an analytical calculation has been conducted as precisely as possible and then this is followed by GEANT4 and MCNPX. Also, the tumor’s depth was included in the present evaluation. The analytical method was based on the expression of $\dot{N}=n({}^{11}B){\Sigma }_{i}\varphi ({E}_{i})\times \sigma ({E}_{i})$in where, $\dot{N}$ is the reaction rate, $n({}^{11}B)$ is the number of the ^11^B isotope and $\varphi ({E}_{i})$ and $\sigma ({E}_{i})$ are the proton’s flux and the cross-section corresponding to the *i*^th^ group of energy respectively. The detailed cross-sections of ${}^{11}B\,(P,\alpha \alpha )\alpha$ have been presented in literature. The Sikora & Weller^46^ reported cross-sections for low energy incoming protons from 600 $keV$ up to 4 MeV and the TENDL 2017^47^ for energy more than 4 MeV. Accordingly, the above expression has been re-written as follow;

1 $$\dot{N}=n({}^{11}B)\times [\{\begin{array}{c}\mathop{\sum }\limits_{i=6}^{40}\varphi ({E}_{i})\times \sigma ({E}_{i})\,\\ \downarrow \\ 600\,keV\le {E}_{i}\le 4\,MeV\end{array}\}+\{\begin{array}{c}\begin{array}{c}\mathop{\sum }\limits_{i=41}^{400}\varphi ({E}_{i})\times \sigma ({E}_{i})\\ \downarrow \end{array}\\ 4\,MeV < {E}_{i}\end{array}\}]$$

**Figure 6:**
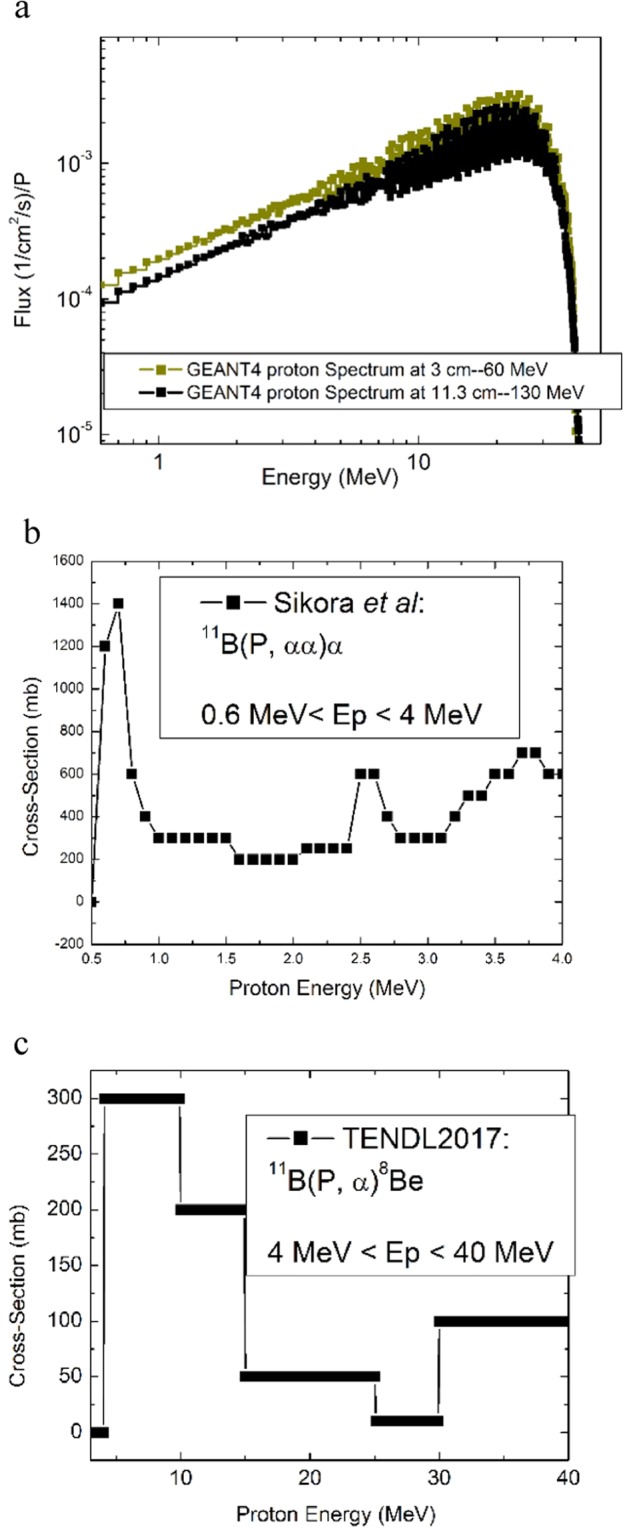
The primary data, necessary for the analytical evaluation of the ${}^{11}B(P,\alpha \alpha )\alpha$ reaction rate; (**a)** the proton spectrum in the tumor at two different depths calculated by GEANT4 to indicate the corresponding fluxes to 400 energy groups, (**b)** the cross-sections re-drawn based on the data reported by Sikora and Weller for the proton energy from 600 $keV$ up to 4 MeV and (**c)** the re-drawn cross-sections based on the TENDL2017 for the protons with the energies more than 4 MeV.

To find the $\varphi ({E}_{i})$ related values, the proton spectra in tumor (at two depth as well) has been calculated by GEANT4 from 600 $keV$ up to the incoming energy presented in Fig. 6a. In this figure, the two proton spectra have been compared for the tumors placed at the SOBP of 1.6 cm to 3 cm and the SOBP of 10 cm to 11.3 cm. From the horizontal axis of this figure, the energy increases by the step of 100 $keV$. The 100 $keV$ bin width means 35 energy groups in the first part of the Eq. 1 and 400 energy groups in the second part. From other hand, the corresponding cross-sections for the two energy ranges have been re-drawn based on the literature. Figure 6b,c represent the two sets of cross-sections; 600 $keV$<Ep<4 MeV and Ep>4 MeV respectively. Notably, at $600\,keV$ energy, the reported value for the cross-section by Sikora & Weller is $1400\,mb$ which, is higher than $1200\,mb$ which has been indicated by Cirrone *et al*.^19^. Finally, using a simple C^++^ programing, the summation of $\varphi ({E}_{i})\times \sigma ({E}_{i})$ for all groups (*i* from 6 to 400) have been calculated precisely according to the two spectra and the corresponding results have been presented as follow;

1a $$\dot{N}=n({}^{11}B)\times \left(70.52\frac{mb}{c{m}^{2}s}\times {10}^{-27}\frac{c{m}^{2}}{mb}\right),{E}_{beam}=45\,MeV\,to\,60\,MeV$$

1b $$\dot{N}=n({}^{11}B)\times \left(63.35\frac{mb}{c{m}^{2}s}\times {10}^{-27}\frac{c{m}^{2}}{mb}\right),{E}_{beam}=121MeV\,to\,130\,MeV$$

**Figure 7:**
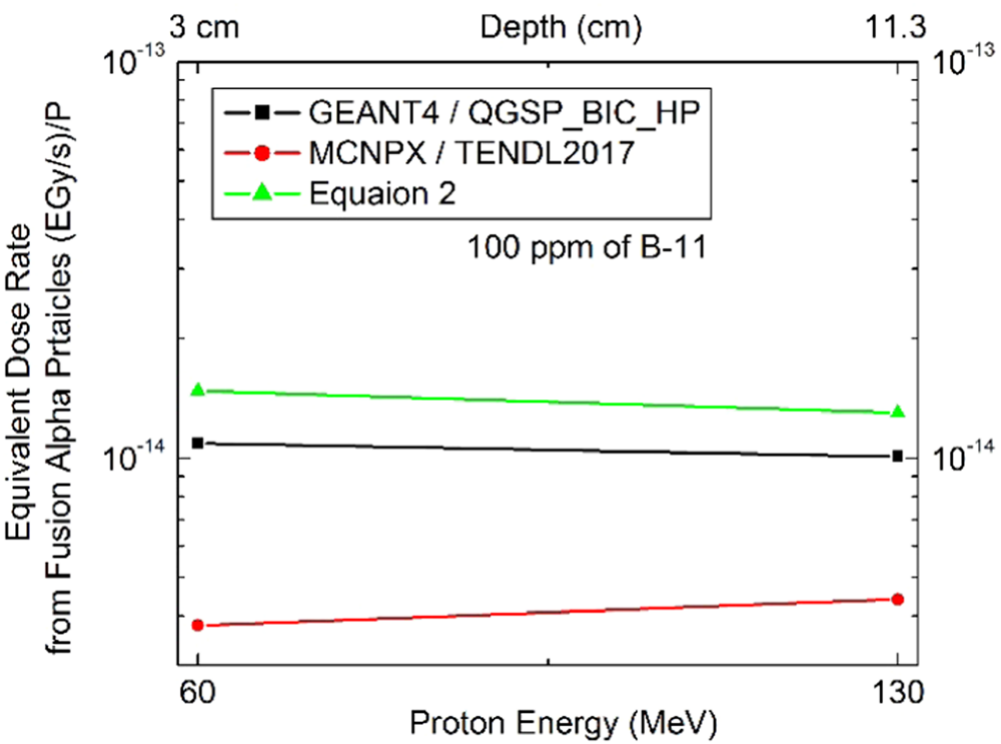
The evaluated dose rate from P-BFT in terms of tumor depth, obtained by analytical method (Eq. 1) and the two MC tools; GEANT4 and MCNPX for 100 ppm of the ^11^B concentration. While the Eq. 1 and GEANT4 presented the higher value than MCNPX, the tumors in different depths almost received similar dose rates.

100 ppm of ^11^B in the tumor with 1.5 cm^3^, leads to the values of $n({}^{11}B)=9\times {10}^{18}$. The total kinetic energy of the fusion alpha particles in ${}^{11}B(P,\alpha \alpha )\alpha$ has the predominant value of 8.67 MeV per reaction. From Eq. 1a and Eq. 1b, and the Eq. 2 (in Method section), the corresponding delivered dose rate fro m the fusion alpha particles has been calculated for 100 ppm of ^11^B and depicted by Fig. 7 accordingly. Also, the MC results have been presented in this figure which shows that, MCNPX has the lower and unreliable results while, the GEANT4 presented more compatible results with the detailed analytical calculations by Eq. 1. This figure, shows that, P-BFT is relatively constant along the tissues depth. The corresponding dose rate obtained in this research is in agreement with Mazzone *et al*. A more quantitative evaluation for P-BFT impact has been performed in the next subsection as the further discussion.

### The comparative discussions

In this section, the dose rate delivered to the tumor by the traditional proton therapy has been calculated based on the two methods for more reliability; using the deposited energy from the protons and Eq. 2 and also using the ICRP conversion factors for protons. Table 3, presents the related data and the normalized dose rate corresponding to the proton particles. Accordingly, each proton entering the tumor causes the dose rate around $\sim {10}^{-9}\frac{Gy}{s}$.

**Table 3:** presents the data to analyze the proton dose rate into tumor using the ICRP flux-to-dose rate conv ersion factors and also by Eq. 2 using energy deposition into tumor.

ICRP				Eq. 2
Proton flux in tumor ($\varphi$)	Conv. Factor for E_p_ less than 60 MeV (${{\rm{C}}}_{{\rm{F}}}$)	Quality factor of proton (${{\rm{Q}}}_{{\rm{f}}}$)	Proton weighting factor (${{\rm{W}}}_{{\rm{F}}}$)	Energy deposited by protons
~0.5 $\frac{p}{c{m}^{2}s}$	2.5 $\frac{mrem/h}{P/(c{m}^{2}s)}$	1.4	5	~14 $(\frac{MeV}{gr})/P$
$\dot{D}=(\varphi \times {{\rm{C}}}_{{\rm{F}}}\times {{\rm{Q}}}_{{\rm{f}}})/{{\rm{W}}}_{{\rm{F}}}$				$\dot{D}=(Eq.2)$
$\dot{D}=\sim 9.6\times {10}^{-10}\frac{Gy}{s}$ or $\sim 4.8\times {10}^{-9}\frac{Sv}{s}$				$\dot{D}=\sim 2.2\times {10}^{-9}\frac{Gy}{s}$

**Figure 8:**
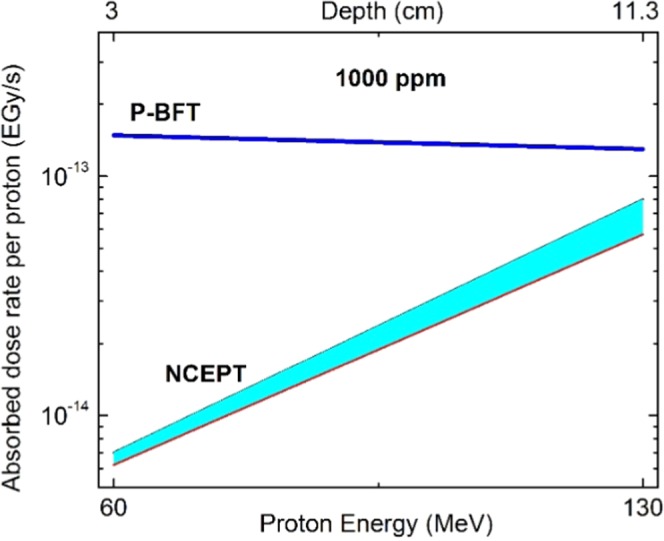
A comparative evaluation for NCEPT and P-BFT in terms of the depth when, 1000 ppm of ^10^B and ^11^B ha s been applied respectively. The P-BFT represents the analytical calculation results while, in NCEPT both the MC results included showing the lower and upper limits. The higher dose corresponding to P-BFT is presented and also, the strong dependency of NCEPT on depth in contrast to P-BFT has been shown.

In the previous subsections, the NCEPT and the P-BFT were re-calculated when they have been applied to the tumor’s in different depths and 100 ppm of the related isotopes, ^10^B and ^11^B respectively. It was found that, both the methods lead to the excess dose rate at least in the order ${10}^{-14}\frac{EGy}{s}$ which in compare to the traditional proton therapy (Table 3) is too low (also reported by Mazzone *et al*. too). To obtain the higher effectiveness from NCEPT and P-BFT, the agent values have been increased to 1000 ppm and the results presented in Figure 8 showing these methods effectiveness, individually and comparative.

This figure shows, both the methods lead to the excess dose rate in the order ${10}^{-13}\frac{EGy}{s}$ which, looking at Table 3, the corresponding dose rate to the traditional proton therapy is still four orders of magnitude higher. It is emphasized that, Cirrone *et al*. and Mazzone *et al*. as examples of the researches in this area have presented their studies based on the ^11^B at much lower level (80 ppm). Therefore, 1000 ppm is only a typical value to make the physical estimation of the highest effectiveness could be approached by these two methods.

From other hand, P-BFT found to have stronger effectiveness than NCEPT as it relies on the proton particles and it was revealed that, P-BFT is not sensitive to the tumor position. While, NCEPT relies on the neutron particles which, are sensitive to the hydrogen concentration (in different organs) and also, the geometry and the position of the treatment area. Nevertheless, these two methods are the motivation for further studies in the development of the proton therapy. For example, searching for the alternative fusion reactions using the non-toxic materials, such as ^15^N(*p, α*)^12^C with (Q = +4.97 MeV) or with the low toxicity substance, such as ^7^Li(*p, α*)α with (Q = +17.35), etc., should be the focus of future investigations.

## Methods

As it was pointed out above, since neither of the methods of P-BFT and NCEPT have been approved in the clinical points of view, the present evaluations are performed theoretically and by means of Monte Carlo (MC) methods. The MC tools, the problem’s setup and the process in obtaining of the results have been introduced according to the following subsections:

### MC Setup

In the present evaluations, for better performance and to obtain the more reliable evaluations, two MC tools have used; MCNPX2.7 and GEANT4.10.5-p02, in which, the MCNPX utilized TENDL 2017^47,48^ and GEANT4 also utilized physics lists of QGSP_BIC_HP^44,45^ for the both proton and neutrons interactions introduced as follow;

TENDL2017: Based on the TALYS Evaluated Nuclear Data Library code up to 200 MEV is used to calculate cross-sections, angular distributions, emitted spectra. Used in proton beam interactions simulations. In this study, addition to the secondary neutron interactions, the proton fusion into ^11^B has been investigated under TENDL2017^47^.

QGSP_BIC_HP: One of the physics lists in GEANT4 in which QGSP is the basic physics list below 10 GeV applying the quark gluon string model stands for hadrons interactions, BIC refers to binary cascade and de-excitation model for hadrons and HP is the high precision model for low energy interactions (<20 MeV) which is used for clinical proton beam with detail neutron transportation.

For evaluating of the proton fusion reaction to ^11^B (P-BFT method), the QGSP_BIC_AllHP was used as the extension of QGSP-BIC-HP physics list which contains the subclass G4ParticleHPInelastic for the low energy charged particles interactions using ENDF/B-VII and TENDL libraries.

### Calculation Methods for NCEPT

Since the NCEPT method is based upon the low energy secondary neutrons production, as discussed in the Results and Discussions Section, they have been evaluated as the range of low energy to fast neutrons (qualitatively) and the related flux as the important parameter in NCEPT method (quantitatively). The neutrons collisions are also calculated to investigate the importance of the tumor’s depth in this method. This evaluation has been performed for the neutron fluxes inside the tumors at two different depths. This will give an overview to the dependency of the NCEPT method on this parameter s. Accordingly, the relatively low depth (SOBP of 1.4 cm to 3 cm) and high depth (SOBP of 10 cm to 11.3 cm) were considered for this purpose. Also, the dose rate to tumor has been calculated based on the 100 ppm of ^10^B concentrations. The evaluations have been performed by QGSP_BIC_HP physics lists for GEANT4 and TENDL2017 libraries for MCNPX as they were introduced in previous subsection. To evaluate the excess dose rate received by tumor, the reaction rate of the n-capture in ^10^B were calculated for the tumor with and without ^10^B agent. In MCNPX, the tally FM card with reaction number of 107 for (n, α) used accordingly. Also the normalized number of the excess produced alpha particles in GEANT4 lead to the reaction rate. To obtain the equivalent dose rate, $\dot{D}$ delivered to the tumor (in terms of $\frac{EGy}{s}$), the following expression were utilized^49^;

2 $$\dot{D}=\frac{\left[Reaction\,Rate\,\left(\frac{1}{{\rm{s}}}\right)\right]\times 2\cdot 3(MeV)\times 1\cdot 6\times {10}^{-13}\left(\frac{{\rm{J}}}{{\rm{MeV}}}\right)}{(tumo{r}^{\mbox{'}}s\,mass)({\rm{kg}})}\,\times ({Q}_{HeavyCharged}=20)$$

in where, *2.3 MeV* is the total kinetic energy deposited from both the alpha (1.5 MeV) and ^7^Li (0.8 MeV) particles released from the n-capture in ^10^B and also the quality factor is 20 for the heavy charged particles^49^ (Table 1).

### Calculation methods for the neutrons absorption dose rate

Another important aspect proposed in NCET method is the reduction of thermal neutron due to capture by ^10^B and, consequently, the reduction of the risk of the secondary neutrons to the patients which has been evaluated as the next step in the Results and Discussions Section. Since the fast neutrons are strongly contributing to dose delivering to the healthy tissues, the equivalent dose rate to healthy tissues contributed by three energy group of the secondary neutrons, namely thermal, epithermal and fast neutrons, have been calculated. To this purpose, the absorbed dose rates from the secondary neutrons were obtained by calculating the neutrons fluxes in healthy tissues and then converting to dose rate using the ICRP-21 flux-to-dose rate conversion factors^50^. The results of neutron dose absorption rate in this study have been reported in terms of both the *Sievert* and *Grays* which this conversion was made using the neutrons weighting factors, (${W}_{R}=\frac{Sv}{Gy}$)^49^.

### Calculation methods for P-BFT

To evaluate the impact of P-BFT in proton therapy, the detailed cross-sections of ${}^{11}B(P,\alpha \alpha )\alpha$ reaction has been considered in an analytical calculation by obtaining the dose rate absorption from the calculated reaction rate. Then, the obtained results were compared to those from the Monte Carlo methods. The analytical calculations were based on the expression 2 (presented in Results and Discussions Section) and have been conducted precisely using the detailed cross-sections presented in literature for all incident protons energy range; from 0.6 MeV up to 4 MeV (using the Sikora reported cross-sections^46^) and from 4 MeV up to 40 MeV (using TENDL2017 cross-section^47^) with the increasing step of 100 keV which means almost 400 energy bins and the cross-sections related to each energy bin have been included in the calculations to perform a precise analytic al evaluation. All calculations were conducted for the tumor at two different depths and 100 ppm of ^11^B concentrations, similar to what was described in the calculation method with the NCEPT.

The MC calculations were performed for proton irradiation of the tumor with and without the ^11^B agent to measure the deposited energy corresponding to the alpha particles. The difference in the measured deposited energy addresses the fusion contribution. This method also has been introduced by Mazzone *et al* which was performed for 80 ppm of ^11^B by performing the simulations for water phantom with a high dosage of ^11^B and without that. Then, the difference in the results for alpha deposited energies have been scaled down to find the corresponding dose rate to 80 ppm. In the present study, the same method has been followed for both the GEANT4 and MCNPX simulations for 100 ppm of ^11^B isotope. As the results validation, the results presented later are in agreements with the work already published by Mazzone *et al*.

### Proton beam adjustment

The present evaluations were based on the proton beam with energy range adjustment according to the Spread Out Bragg Peak (SOBP) concept for a tumor with the diameter of 1.4 cm. This beam adjustment was considered for all reactions corresponding to both the NCEPT and P-BFT methods. The beam diameter also was 2 mm.

### ^10^B and ^11^B concentration values

In this research, the results were based on the using of 100% enriched ^10^B for NCEPT and ^11^B for P-BFT. Also, the mass concentrations of 100 ppm were applied as it is near the concentration of 80 ppm discussed by Cirrone *et al*.^19^ and Mazzone *et al*.^45^ and most of the other performed works^3–8^. The higher values up to 1000 ppm in this research have been examined for a physical evaluation of the impacts regarding the NCEPT andP-BFT as discussed at the end of the Results and Discussions section. Though, in the work performed by Safavi-Naeini *et al*.^9^, 3000 ppm has been suggested for the agent, its feasibility must be studied in technical and clinical points of views.

### Important parameters

The information about the problem’s parameters and the hypotheses related to the present calculations are summarized in Table 1.

## Conclusions

A comparative study has been conducted between the two recently proposed methods for the enhancement of radiation effectiveness in proton therapy, P-BFT and NCEPT. The comparison aims to investigate their effectiveness and weaknesses. While the proton fusion therapy (P-BFT method) was based on the excess dose rate transfer to the tumor site using the boron-11 agent to stimulate $p+{}^{11}B\to 3\alpha$ fusion reaction, the exclusive use of boron-10 atoms (in NCEPT method) will lead to another process of using the intrinsic thermal neutrons captured by boron-10 during the ongoing proton therapy resulting in excess dose rate transfer to the treatment area. The comparison based on the both GEANT4 and MCNPX simulation revealed that the NCEPT is a depth dependent method. In a similar situation (the same concentration of agents), the impac t of NCEPT method is much lower compared to P-BFT, particularly for the tumors at lower depths. Although the excess dose rate from both the methods are about more than several orders of magnitude lower than the dose rate from proton beam therapy even when using 1000 ppm of boron. However, the important advantage of using the NCEPT method was the proposed decrease in the thermal neutrons by n-capturing reaction with ^10^B that leads to a significant decrease in the thermal neutron dose rate to healthy tissues. Nonetheless, the thermal neutrons risk reduction is heavily dependent upon the concentration of ^10^B agent. The results of our study reveal that this advantage is not accessible, since the level of ^10^B needs to be so high (tens of thousands ppm) to make it a significant impact. These two innovative methods should motivate further investigation on newer radiation therapies in the treatment of cancer. As our studies suggest the application of alternative fusion reactions individually or combined is worth pursuing in the future clinical trials.

## Data Availability

The datasets generated and/or analyzed during the current study are available from the corresponding author.
